# Middle ear neuroendocrine tumor: a case report

**DOI:** 10.1093/jscr/rjac257

**Published:** 2022-06-17

**Authors:** Azeddine Lachkar, Drissia Benfadil, Fahd Elayoubi

**Affiliations:** ENT and Head and Neck Surgery Department, University Hospital Center Mohammed VI, Faculty of Medicine and Pharmacy, Mohammed First University, Oujda, Morocco; ENT and Head and Neck Surgery Department, University Hospital Center Mohammed VI, Faculty of Medicine and Pharmacy, Mohammed First University, Oujda, Morocco; ENT and Head and Neck Surgery Department, University Hospital Center Mohammed VI, Faculty of Medicine and Pharmacy, Mohammed First University, Oujda, Morocco

## Abstract

Neuroendocrine tumors are extremely rare in the middle ear. These tumors represent a spectrum of tumors with a diverse range of molecular abnormalities, functionality and anatomical locations. We present a rare case of middle ear neuroendocrine tumor, review the pathology and differential diagnosis of the tumors, and discuss the management and follow-up of patients with these tumors. We suspect that the middle ear neuroendocrine tumor is underdiagnosed and more cases can be detected through education and personal experience. Treatment is surgical resection, and long follow-up is recommended.

## INTRODUCTION

Neuroendocrine tumors represent a spectrum of tumors with a diverse range of molecular abnormalities, functionality and anatomical locations. Middle ear neuroendocrine tumors represent <2% of primary ear tumors [1].

They raise significant questions as to the provenance of such tumors arising from an area not generally known to harbor neuroendocrine tissue, the classification, etiology and biological characteristics of these diseases are not clear. The existing research tends to believe that it is a kind of low-grade malignant lesion with mild cell morphology and local recurrence, but there is still a great controversy about the existence of distant metastasis [2].

In this paper, we present a rare case of middle ear neuroendocrine tumor, review the pathology and differential diagnosis of the tumors, and discuss the management and follow-up of patients with these tumors.

## CASE PRESENTATION

**Figure 1:**
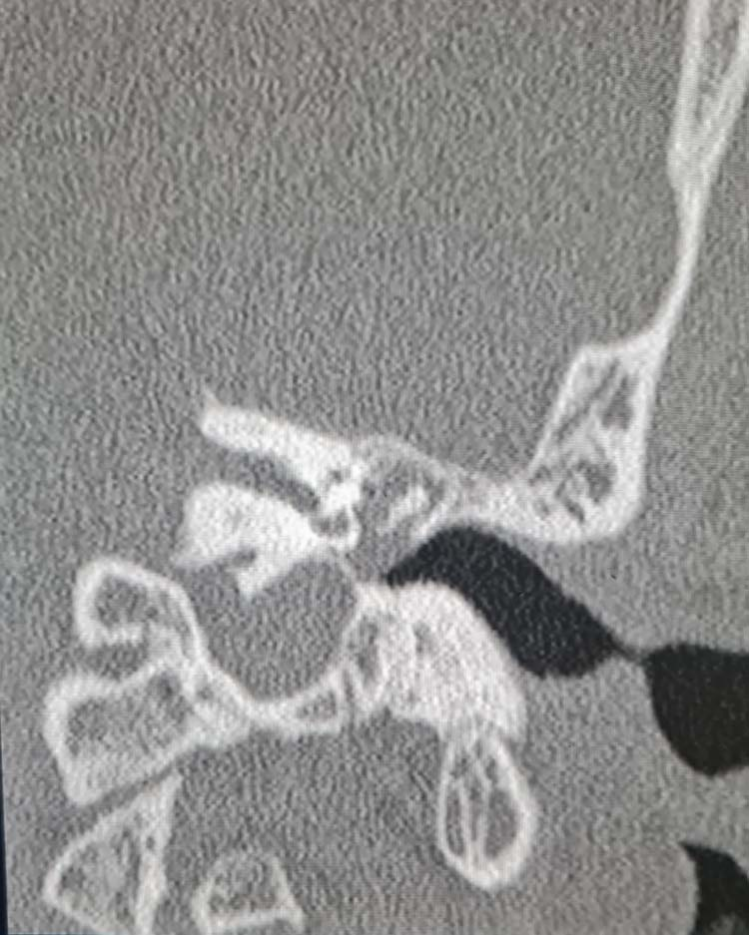
Coronal computed tomography image showing the mass with soft tissue density filling the middle ear cavity.

A 35-year-old male with no medical history, presented to us with gradual left ear hearing loss and ear fullness over 1-year duration. There was no prior history of trauma or exposure to unusually loud noise and no notion of family deafness. A physical examination showed a normal left external auditory canal and the tympanic membrane was bulging and thickened. Audiometry objectified conductive moderate hearing loss. A contrast-enhanced computed tomography scan was performed revealing a mass with soft tissue density. The mass localized into the middle ear with extent to the attic, pushing and dislocating the ossicles, without evidence of bone invasion (Fig. 1).

**Figure 2:**
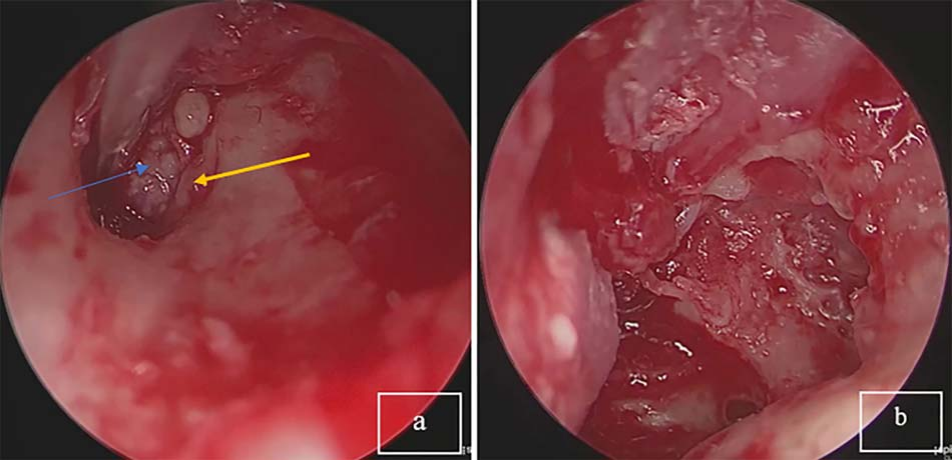
(**a**) Tumor mass in the eardrum, blue arrow: tumor; yellow arrow: corda tympani. (**b**) Complete resection of the tumor.

An endoscopic exploratory tympanotomy was performed. During the procedure, we found a bony mass filling the atrium. The bony structure was free from the surrounding walls of the middle ear, it was found that the stapes was absent, and descending branch of the incus was lysed. A polypoidal mass filling the attic. A complete surgical excision of the mass was performed endoscopically (Fig. 2). After the removal of the mass, a tympanoplasty type III was performed using a chondral perichondrial graft from tragus.

**Figure 3:**
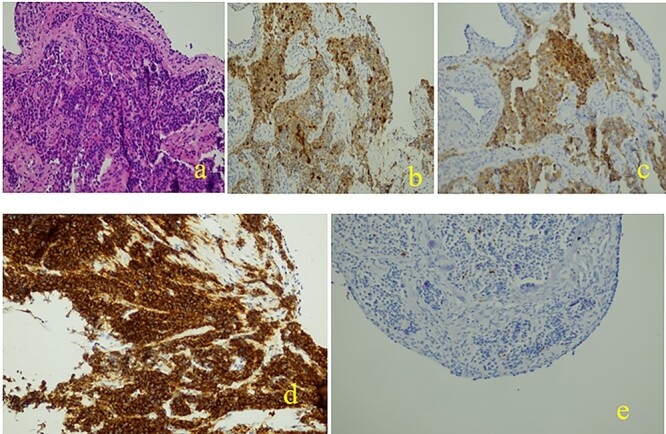
Histopathological and immunohistochemical examinations: (**a**): Neoplastic cells are organized to great extent in crowded glandular formations, while surrounding stroma is characterized by fibrosis and hyalinosis, (**b**): chromogranin +, (**c**): synaptophysin +, (**d**): cd56 +, (**e**): ki67 +.

An immunohistochemical evaluation revealed positivity for AE1/AE3, chromogranin A, synaptophysin and CD56. The evaluated proliferation index of the anti-K67 antibody is estimated at 2%. The final diagnosis was well-differentiated Grade 1 neuroendocrine tumor (Fig. 3). The evolution was simple.

## DISCUSSION

Middle ear neuroendocrine tumors are extremely rare, mainly with benign characteristics. They are also called middle ear adenomas, the terminology adenoma is not appropriate, as these tumors can be invasive and even occasionally metastatic. Sylvia L. Asa has shown that these neuroendocrine tumors are usually of low proliferative grade, similar to Grade 1 tumors of the gastroenteropancreatic tract, but they occasionally have Ki67 labeling indices that would allow classification as Grades 2 and 3 neuroendocrine tumors [3].

The most frequent symptoms are hearing loss, which is commonly of the conductive type, ear fullness, tinnitus and/or ear pain. Rarely, they may present with facial nerve palsy [4].

The differential diagnosis of middle ear neuroendocrine tumors is extensive and includes Jugulotympanic paraganglioma, acoustic neuroma, meningioma, rhabdomyosarcoma and adenocarcinoma. However, the radiographic, histologic and immunohistochemical features help to distinguish them from other entities [5].

On the computed tomography, the tumor is characterized by soft tissue density in the mastoid bone without blood vessels, which can be extended to the middle ear and mastoid. The ossicular chain is usually embedded into the tumor, but there is no bone erosion [2].

The histopathology and immunohistochemistry not only confirms the diagnosis of the neuroendocrine tumors, but also plays an important role in grading and histopathological staging. The tumor cells express positivity for AE1/3 and for CK7, as well as positivity for general neuroendocrine markers, including chromogranin A, synaptophysin and CD56. The Ki-67 proliferation index may play an important role in prognostication and therapeutic decision. The other most important factor determined by the histology is the degree of differentiation of the cells [4].

Complete surgical resection is the treatment of choice. Most patients undergo tympanomastoidectomy with microsurgical removal of the tumor, when external auditory canal, or mastoid are involved. When there is adhesion to the ossicles, they are removed, and the chain is reconstructed when it is necessary. In our case, a complete resection was performed endoscopically. Adjuvant treatment is not recommended but long-term follow-up is recommended due to slow progression and difficulty to resect with wide margins [6, 7].

## CONCLUSION

Middle ear neuroendocrine tumors are a rare disease entity. The etiology of these tumors is not clear and their classification is not well defined. Further studies on the behavior of this neuroendocrine tumors subtype and its response to the standard treatment modalities are needed.

## DATA AVAILABILITY

The patient’s data are available upon reasonable request to the corresponding author.

## CONFLIT OF INTEREST STATEMENT

None declared.

## FUNDING

None.
